# ASD-SWNet: a novel shared-weight feature extraction and classification network for autism spectrum disorder diagnosis

**DOI:** 10.1038/s41598-024-64299-8

**Published:** 2024-06-13

**Authors:** Jian Zhang, Jifeng Guo, Donglei Lu, Yuanyuan Cao

**Affiliations:** 1 School of Internet of Things and Artificial Intelligence, Wuxi Vocational College of Science and Technology, Wuxi, 214028 China; 2 College of Computer Science and Engineering, Guilin University of Aerospace Technology, Guilin, 540004 China

**Keywords:** Computer science, Biomedical engineering, Cognitive neuroscience

## Abstract

The traditional diagnostic process for autism spectrum disorder (ASD) is subjective, where early and accurate diagnosis significantly affects treatment outcomes and life quality. Thus, improving ASD diagnostic methods is critical. This paper proposes ASD-SWNet, a new shared-weight feature extraction and classification network. It resolves the issue found in previous studies of inefficiently integrating unsupervised and supervised learning, thereby enhancing diagnostic precision. The approach utilizes functional magnetic resonance imaging to improve diagnostic accuracy, featuring an autoencoder (AE) with Gaussian noise for robust feature extraction and a tailored convolutional neural network (CNN) for classification. The shared-weight mechanism utilizes features learned by the AE to initialize the convolutional layer weights of the CNN, thereby integrating AE and CNN for joint training. A novel data augmentation strategy for time-series medical data is also introduced, tackling the problem of small sample sizes. Tested on the ABIDE-I dataset through nested ten-fold cross-validation, the method achieved an accuracy of 76.52% and an AUC of 0.81. This approach surpasses existing methods, showing significant enhancements in diagnostic accuracy and robustness. The contribution of this paper lies not only in proposing new methods for ASD diagnosis but also in offering new approaches for other neurological brain diseases.

## Introduction

Autism spectrum disorders (ASD) constitute a complex and diverse group of neurodevelopmental disorders, renowned for their extensive range of social interaction and communication challenges, as well as characteristic stereotypical and repetitive behaviors^1^. Neurodiversity is considered one of the key factors contributing to autism. Latest statistics from the Centers for Disease Control show that the incidence rate of ASD in the U.S. is now one in every 54 individuals, signifying a substantial health and socio-economic burden on society and families^2,3^. Individuals with ASD often face challenges related to communication and interaction, negatively impacting not only their own quality of life but also affecting their families, education, and social relationships^4^.

In recent years, the application of machine learning methods in the medical field has shown tremendous potential, offering new opportunities for early diagnosis and precision medicine^5^. Kang et al.^6^ utilized transfer learning from pre-trained deep convolutional neural networks for robust feature extraction from MRI images, followed by the application of machine learning classifiers for accurate tumor classification. Ullah et al.^7^ employed median filtering and contrast limited adaptive histogram equalization for preprocessing, followed by feature extraction using 2D discrete wavelet transform and feature reduction using color moments. Finally, a feed-forward neural network classified the MRI images. They presented a novel method for brain tumor segmentation using a cascade multiscale residual attention U-Net architecture, emphasizing enhanced accuracy through focused learning mechanisms and post-processing refinement, demonstrating improved performance on specific tumor regions^8^.

Within the field of ASD diagnostic approaches, a variety of machine learning methods including support vector machines (SVM)^9^, random forest algorithms^10^, autoencoders (AE)^11^, single layer perceptrons (SLP)^12^, and deep neural networks (DNN)^13^ have garnered significant interest and have been the focus of extensive research^14^. Chen et al.^15^ conducted a study on functional connectivity analysis in ASD, utilizing various machine learning techniques, including SVM, to identify biomarkers for ASD^16^. This study highlighted the importance of connectivity data in ASD classification. Heinsfeld et al.^17^ employed machine learning techniques and transfer learning^18^, using AEs for feature extraction and DNNs for classification, to identify ASD patients. They supported their research with the ABIDE dataset. In these studies, researchers utilized large-scale brain imaging datasets and successfully established diagnostic models through feature selection and classification techniques, enhancing the accuracy of early ASD diagnosis^19^. Neural network methods like SLP and DNN have also been used to process multimodal data in ASD, improving pattern recognition performance and predictive accuracy^20^. These research efforts have opened new possibilities for the application of machine learning methods in ASD diagnosis, providing powerful tools to improve the quality of life for ASD patients.

Despite the significant advancements in diagnosis of ASD using machine learning methods, deep learning approaches, particularly various convolutional neural networks (CNN) and graph convolutional networks (GCN)^21^, are spearheading developments in the field of ASD research. Deep learning methods, through multilayered feature extraction and pattern recognition, hold promise for more profound identification of ASD biomarkers and enhanced diagnostic precision. A recent study by Mendes et al.^22^ utilized 3D CNNs to process brain imaging data of ASD patients, particularly in the areas of structural magnetic resonance imaging and functional magnetic resonance imaging (fMRI). Researchers employed deep learning techniques, including 3D CNNs and transfer learning, for brain image feature learning and classification, thereby improving the accuracy of early ASD diagnosis. Additionally, GCNs, a deep learning method specifically designed for graph data, have been applied in ASD research for analyzing brain network connections and social network data^23^. These methods enable researchers to understand more comprehensively the relationships between ASD, brain connectivity, and social interactions, providing new insights for early diagnosis and intervention^24^. This body of work demonstrates the potential of deep learning approaches in the ASD domain, particularly in integrating multimodal and graph data analyses. The application of deep learning technologies offers deeper insights and aids in enhancing the accuracy of early ASD diagnosis and the realization of precision medicine^25^.

We have developed a network model called ASD-SWNet, which encompasses fMRI data processing, data augmentation, and feature extraction, and on this basis, we have proposed an efficient CNN framework to complete the task of automatic diagnostic classification. The key contributions of this study can be summarized as follows:

- An unsupervised feature extraction method and a supervised learning convolutional neural network classification method have been proposed. The shared weights approach effectively combines the AE and CNN. The CNN effectively discerns more significant features from the low-dimensional data obtained by the AE, which leads to a reduction in the model’s complexity and an enhancement in its overall performance.
- A convolutional neural network model tailored for ASD diagnosis has been designed to extract high-level features and complete the classification task. Our custom-designed CNN adapts better to the characteristics of ASD data, and the judicious design of convolutional layers, batch normalization, and Dropout significantly enhances the model’s feature extraction capability. The appropriate selection of activation functions also contributes to the improved performance of the model.
- In order to solve the problem of insufficient time series data such as ASD, an improvement was made to a known data augmentation algorithm. This moderate level of data augmentation has been verified to improve the model's performance and enhance its robustness, generalization ability, and noise resistance.
- The ASD-SWNet framework we have developed was rigorously tested on two publicly available ASD datasets and benchmarked against current advanced methods. The outcomes of both nested ten-fold cross-validation^26^ and leave-one-out cross-validation (LOOCV)^27^ validate the efficiency of our proposed approach in improving classification accuracy.

## Materials and methods

### Datasets and data preprocessing

All research in this manuscript complies with all relevant ethical regulations. The online available ABIDE dataset is utilized in this study. Ethical approval was not required as confirmed by the license attached with the open-access data, since they were previously approved by each site’s local IRB. All data were obtained with informed consent from the subjects. To gain a deeper understanding of the neural mechanisms underlying ASD, the ABIDE dataset^28^ compiles brain MRI data from ASD patients and healthy developing children (HC). The dataset and preprocessed information used in this study are shown in Table 1.

**Table 1:** Dataset and preprocessing information.

Dataset	Preprocessing pipeline	ASD	HC	Acquisition
ABIDE-I	Configurable pipeline for the analysis of connectomes	419	452	https://fcon_1000.projects.nitrc.org/indi/abide/abide_I.html
ABIDE-II	Data processing assistant for resting-state fMRI	76	102	https://fcon_1000.projects.nitrc.org/indi/abide/abide_II.html

As shown in Table 1, we preprocess the ABIDE data using the configurable pipeline for the analysis of connectomes (CPAC)^29^. Hence, we preprocess the ABIDE-I data using CPAC, discarding samples with anomalies or missing time series. We ultimately utilize data from 871 subjects in ABIDE-I, including 419 ASD individuals and 452 HC. The data processing assistant for resting-state fMRI (DPARSF)^30^ is employed for preprocessing the ABIDE-II data, selecting 76 ASD patients and 102 HCs from various sites. To mitigate potential biases from the site, age, gender, and other phenotypic factors, our study ensures a balanced distribution among the 178 participants.

Functional connectivity (FC) is employed to generate features from the time series of rs-fMRI brain imaging data. We approximate the FC between two brain regions using the Pearson correlation coefficient, which is a reliable indicator of linearity between two-time series, H and K, given a length $T$, represented as:

1 $$\rho uv = \frac{{\sum\limits_{t = 1}^{T} {\left( {ut - \overline{u}} \right)} \left( {vt - \overline{v}} \right)}}{{\sqrt {\sum\limits_{t = 1}^{T} {\left( {ut - \overline{u}} \right)^{2} } } \sqrt {\sum\limits_{t = 1}^{T} {\left( {vt - \overline{v}} \right)^{2} } } }}$$

where $\overline{v}$ and $\overline{u}$ are the average values of time series H and K, respectively.

The functional connectivity matrix is computed from the Pearson correlation, which has symmetrical upper and lower triangular values, with the main diagonal representing the self-correlation of each brain region. Therefore, we exclude the values from the lower triangle and the main diagonal, retaining only the upper triangular values and flattening them into a one-dimensional vector. In our study, we use the CC200 atlas, which divides the brain into 200 regions of interest (ROI), According to Eq. (2), a total of 19,900 features are generated.

2 $$S = \frac{n(n - 1)}{2}$$

where $n$ represents the number of ROIs.

Subsequently, we apply the Recursive Feature Elimination (RFE) method^31^ to select the subset of features most beneficial to model performance. RFE iteratively constructs the model, removing the least impactful features during each iteration until the feature count is reduced to 2000. This approach aids in identifying the most crucial features, reducing redundancy, enhancing the model's generalizability, and lowering computational costs.

### Improved data augmentation algorithm

The performance of the model is significantly influenced by both the size and the quality of the dataset. To address the challenges of limited and imbalanced data samples, this study improved the data augmentation algorithm proposed by Eslami et al.^32^.

The inspiration for the data augmentation algorithm comes from the synthetic minority oversampling technique^33,34^. This algorithm creates new data in the feature space by utilizing the nearest neighbor of the sample. First, find K-nearest neighbor $\left\langle {\hat{x}1,\hat{x}2,...,\hat{x}k} \right\rangle$ of sample $x$. Then, we randomly select a sample $\left\langle {\hat{x}i} \right\rangle$ from the K-nearest neighbor, and use Eq. (3) to generate a new feature vector $x^{\prime}$:

3 $$x^{\prime} = x + \varphi \left( {\hat{x}i - x} \right)$$

where $\varphi$ is the threshold value, which is a random number between [0,1]. Generally, the K-nearest neighbor of sample $x$ is calculated based on Euclidean distance. However, the Euclidean distance can only reflect the overall relationship between sequences, rather than the local changes^35^. To find the K-nearest neighbor, we introduce principal component analysis (PCA) ^36^ of the extended Frobenius norm (EROS)^37^.

First, estimations are made for the covariance matrices of two multivariate time series (MTS), and then their eigenvalues and eigenvectors are calculated. Finally, the similarity of each MTS element is measured by the eigenvalues obtained in MTS. The EROS distance between MTS A and B is calculated, as shown in Eq. (4):

4 $$DEROS(A,B,\omega ) = \sqrt {2 - 2\sum\limits_{i = 1}^{n} {\omega i\left| { < ai,bi > } \right|} }$$

where $\omega$ is the weight vector matrix. The singular value decomposition is performed on the covariance matrices of A and B to obtain the eigenvectors $VA = [a1,a2,...,an]$ and $VB = [b1,b2,...,bn]$. In Eq. (4), $ai$ and $bi$ are orthogonal column vectors with length n. Equation (4) can be further simplified into Eq. (5):

5 $$DEROS(A,B,\omega ) = \sqrt {2 - 2\sum\limits_{i = 1}^{n} {\omega \left| {\sum\limits_{j = 1}^{n} {aibi} } \right|} }$$

The key operation of PCA is to simplify the original data. For MTS A and B, the principal elements of each matrix are obtained first, and then z principal elements are selected. The similarity among z principal elements is calculated by Eq. (6):

6 $$SPCA\left( {A,B} \right) = \sum\limits_{i = 1}^{z} {\sum\limits_{j = 1}^{z} {\cos^{2} \theta ij} }$$

where $\theta ij$ is the angle between the $i{\text{th}}$ pivot of A and the $j{\text{th}}$ pivot of B. Then, the similarity of EROS of A and B is defined as Eq. (7):

7 $$EROS(A,B,\omega ) = \sum\limits_{i = 1}^{n} {\omega i\left| {cos\theta i} \right|}$$

where $\theta i$ is the angle between $ai$ and $bi$. $\omega$ is the weighted vector of the eigenvalues of the MTS dataset. First, the eigenvalues in each MTS are normalized, and subsequently, all the eigenvalues in the whole dataset are normalized by the aggregation function, such that $\sum\limits_{i = 1}^{n} {\omega i} = 1$.

The dimension of the covariance matrix for each sample is $n \times n$, where $n$ is the number of ROIs of the atlas. The covariance matrix of each dataset is calculated in advance. Research conducted by Eslami et al.^38^ on ADHD indicated that EROS effectively measures similarity in fMRI data. PCA of EROS can be applied to determine the K-nearest neighbor distance. In our approach, after identifying the K-nearest neighbor for each sample in the training set and randomly choosing one, we utilize linear interpolation between the selected sample and its nearest neighbor to produce new samples. This algorithm is used to enhance the data. A composite sample is created for each data in the dataset and placed into the dataset. By setting different augmentation factor $\gamma$, the size of the dataset is increased to $\gamma$ times the original. The pseudocode of our proposed data augmentation algorithm is shown in Algorithm 1.

**Algorithm 1:**
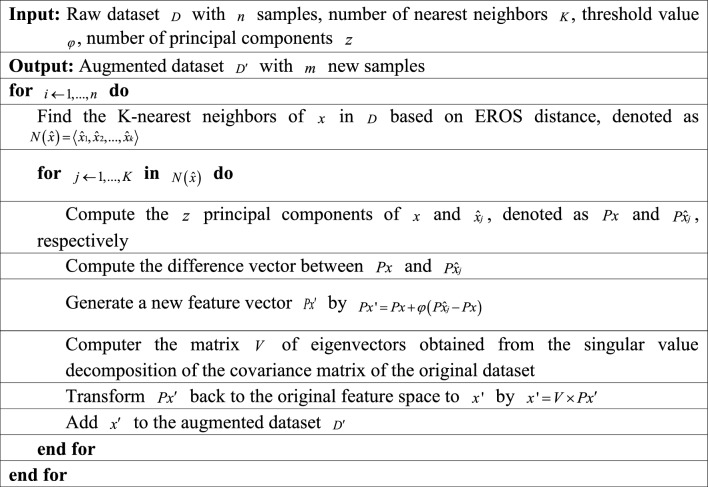
Data augmentation pseudocode.

### Unsupervised learning feature extraction method

Autoencoders, as an unsupervised learning method^39^, aim to reduce dimensionality and extract features by learning compressed representations of data. Their fundamental design includes an encoder, a bottleneck layer, and a decoder. The encoder transforms the input data into a lower-dimensional latent space, as depicted by the bottleneck layer. Subsequently, the decoder reverts this latent representation to the original data space, aiming to reduce the reconstruction error. The denoising autoencoder (DAE) is a variation of the standard autoencoder, designed to learn robust representations by introducing noise into the input data. The DAE undergoes training to reduce the discrepancy between the input data with added noise and the decoder’s output, compelling the model to acquire feature representations that are resilient to noise. This training process enables the DAE to extract features that are resilient to variations in the data, enhancing the model’s robustness and generalizability. In our study, the DAE is utilized for low-level feature extraction from data and also to reduce the feature dimensions to a manageable range.

**Figure 1:**
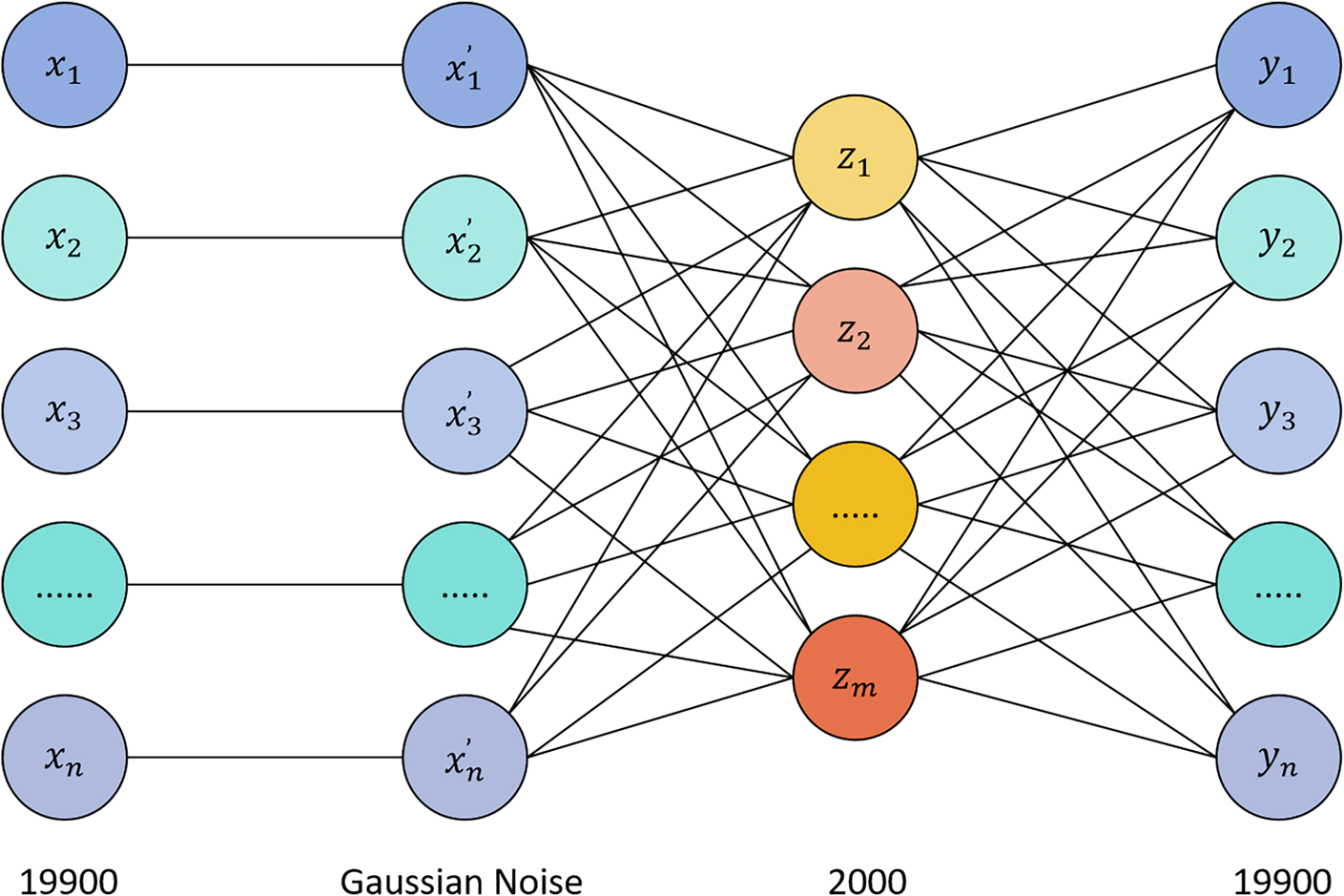
Denoising autoencoder with Gaussian noise.

There are many ways to set noise in DAEs. To retain more information from the original data and simulate random errors in the data, in this study, we add Gaussian noise to the preprocessed data $x$ to obtain a new data representation $x^{\prime}$. Eq. (8) shows how Gaussian noise is added to the original data:

8 $$x^{\prime} = x + \varepsilon$$

where $\varepsilon$ signifies random noise drawn from a Gaussian distribution, characterized by a mean of 0 and a standard deviation of $\sigma$. The mean squared error loss function is utilized to determine the error between the original input $x$ and the reconstructed input $y$, with the aim of training the autoencoder to minimize this reconstruction error. In the case of the CC200 atlas, the encoder and decoder were configured with 19,900 nodes each, and the bottleneck layer was allocated 2000 nodes, facilitating a reduction in dimensionality. Our DAE model designed for unsupervised feature extraction is shown in Fig. 1.

### Supervised learning with convolutional neural networks

**Figure 2:**
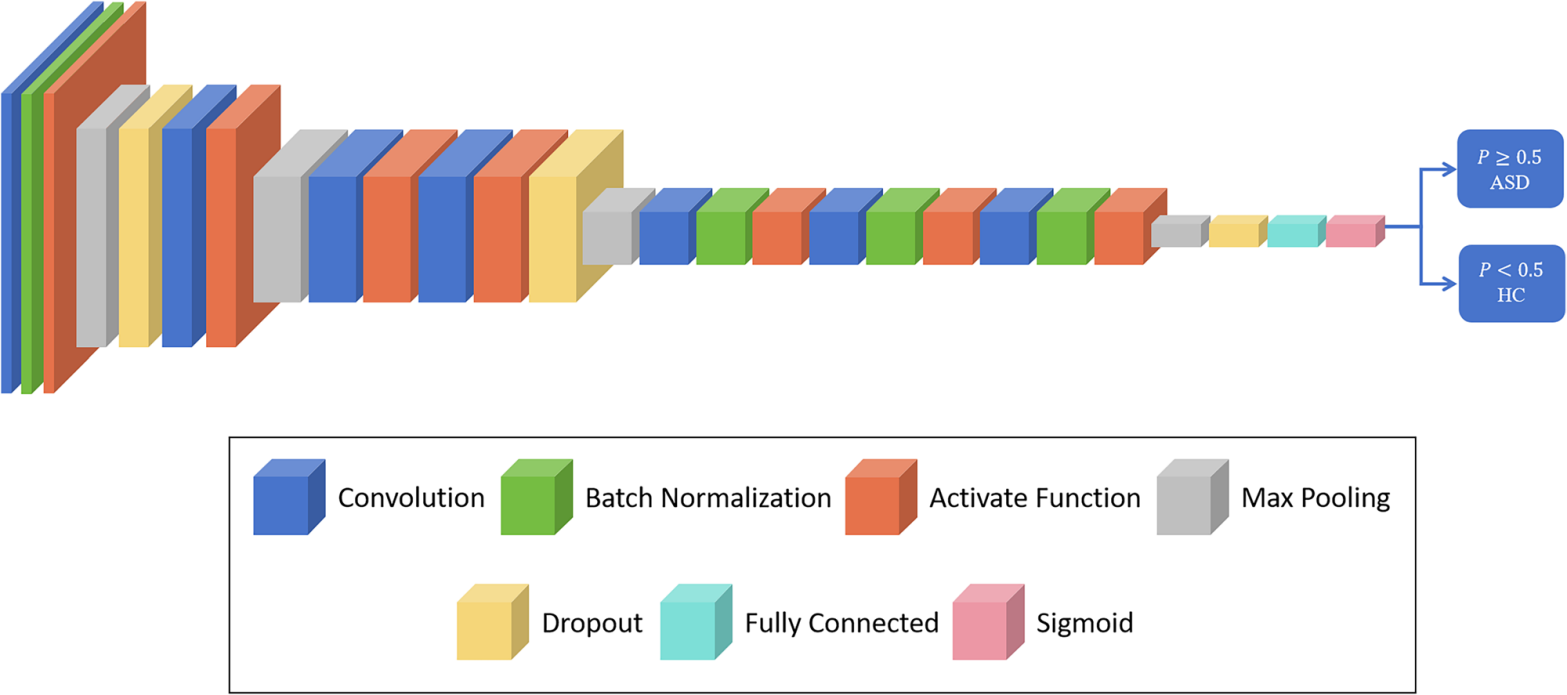
Supervised learning classification convolutional neural network model framework.

Convolutional neural networks can extract high-level features from data and classify samples. However, the latest network models generally have many layers, which is not suitable for the characteristics of the ASD dataset. Therefore, we designed a convolutional neural network model for the automatic diagnosis of ASD, which involves convolutional layers, batch normalization (BN), activation function layers, max pooling, dropout, fully connected layers, and a sigmoid activation function. Our proposed classification model framework is shown in Fig. 2.

CNN extracts a set of high-level features from the low-level features generated after DAE feature extraction. Convolutional layers apply convolution operations to feature maps through kernels, automatically learning and extracting features from the input data.

Batch Normalization^40^ is typically applied after convolution operations. BN enhances the training stability of the network, accelerates convergence, and improves model generalizability. However, BN requires the computation of mean and variance in each batch and introduces scaling and shifting parameters, increasing computational overhead and network complexity. Therefore, in our designed CNN model, BN layers were not added in the middle three convolution operations.

Max pooling layers are a key component of our designed CNN network model, with one of their primary functions being feature dimensionality reduction. In addition, max pooling layers select the most prominent feature values in each region, extracting important information from the original data, and enhancing the model's generalization ability and noise resistance. Moreover, max pooling layers contribute to reducing the risk of overfitting by simplifying the model through dimensionality reduction, particularly vital for tasks with limited medical imaging data.

Dropout layers randomly set the output of a portion of neurons to zero during training, serving as an effective regularization technique. The primary goal of Dropout is to mitigate the risk of overfitting, forcing the model not to rely on any single neuron. It helps reduce the coupling between neurons, thereby enhancing the model’s robustness.

The Sigmoid function^41^ serves as the activation function for the output layer, facilitating binary classification and mapping the network's output to values indicative of probabilities.

### Shared weights network framework

**Figure 3:**
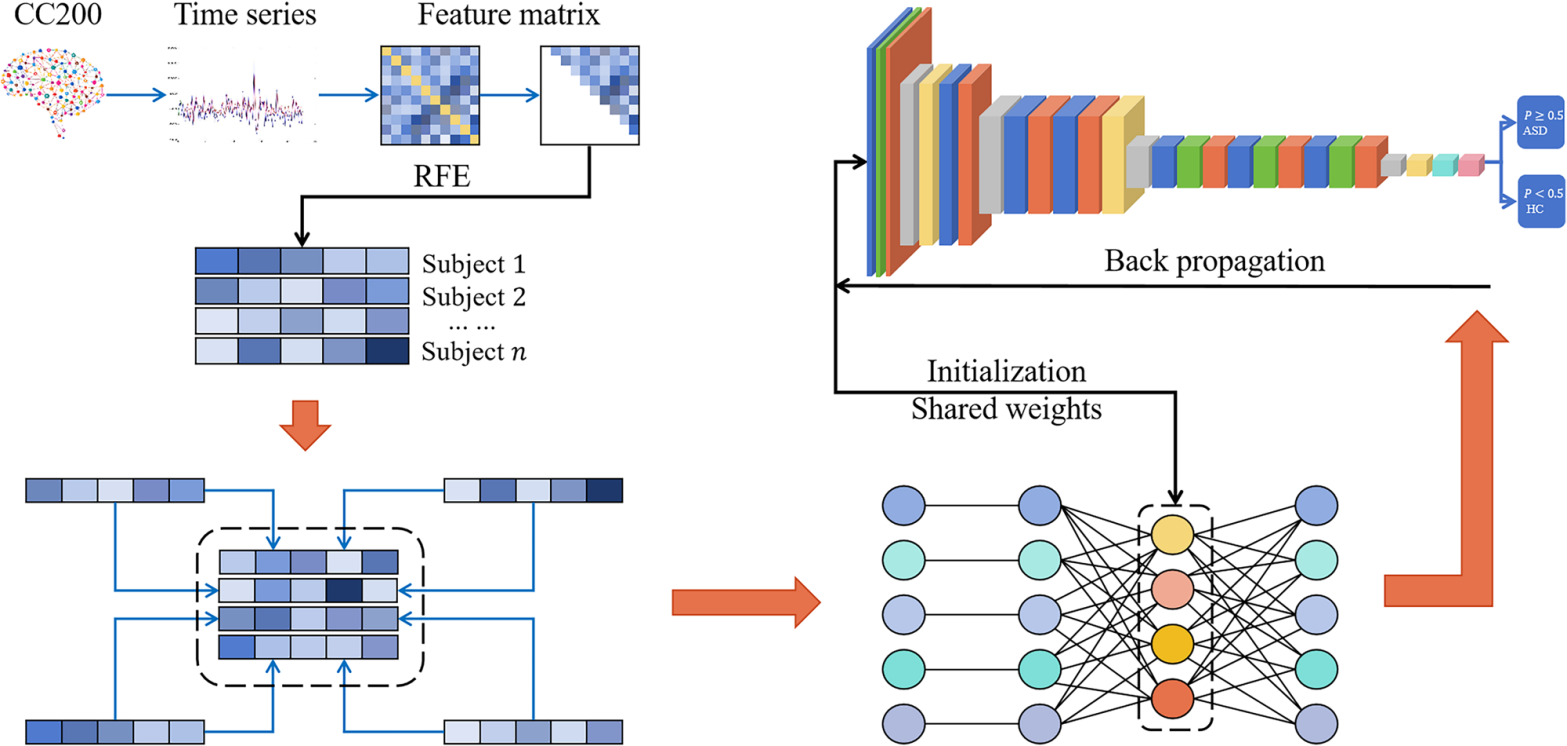
The proposed overall network framework of ASD-SWNet.

We have constructed the feature extraction method for unsupervised learning and the convolutional neural network for supervised learning. Next, we implement weight sharing between AE and CNN. Specifically, we construct an autoencoder (Fig. 1) and train it on input data in an unsupervised manner to minimize reconstruction error so that the decoder output is as close as possible to the input data. The training process uses standard backpropagation algorithms and mean squared error loss functions. The feature representation extracted from the bottleneck layer of AE is used for the subsequent CNN input. We build a convolutional neural network model (Fig. 2) and connect the entire model (comprising convolutional layers, fully connected layers, and the bottleneck layer of the autoencoder) to form an end-to-end model. Once AE training is complete, we initialize the weights of its bottleneck layer as part of the CNN, thus achieving weight sharing. Meaning, that the feature representation acquired by the AE is utilized to initialize the weights of the convolutional layers in the CNN. This is intended to align CNN’s initial feature representation more closely with the useful characteristics of the data. Subsequently, the low-dimensional feature representation obtained from the AE bottleneck layer is input into the model to start joint training of the CNN model, including the shared weight AE and additional CNN layers. Throughout the training phase, the primary objective of the model is to minimize the cross-entropy classification loss function. We use backpropagation algorithms to update the weights of the shared weight autoencoder part and CNN layers to reduce classification loss. Through backpropagation and gradient descent optimization algorithms, we update the entire model’s weights to achieve optimal performance in classification tasks. Our proposed ASD-SWNet method for diagnosing autism spectrum disorder with shared weights is shown in Fig. 3.

## Experimental results and discussion

### Experiment settings

The experiments of this study are primarily conducted on the ABIDE-I dataset using nested ten-fold cross-validation and on the ABIDE-II dataset using leave-one-out cross-validation. Nested ten-fold cross-validation is executed on two levels to overcome bias in model selection and performance evaluation. ASD-SWNet hyperparameter settings and CNN configurations are shown in Tables 2 and 3. All models were developed using the open-source machine learning library PyTorch and experiments were conducted on a GeForce GTX 4060 GPU. For performance evaluation^42^, accuracy, precision, recall, F1-score, and the area under the ROC curve (AUC) were used as metrics.

**Table 2:** ASD-SWNet configurations.

Parameter name	Parameters
Cross-validation fold	10
RFE	2000
K-nearest neighbor	5
Augmentation factor	2
DAE optimizer	Stochastic gradient descent (SGD)
SGD momentum	0.9
Learning rate	0.0001
Gaussian noise	0.1
Epoch	200
Dropout rate	0.3
Early stopping	20
CNN optimizer	Adaptive optimizer (Adam)

**Table 3:** CNN configurations.

Layer	Settings	Output shape
Input		(2000)
Conv 0	Kernel size: 7 Filters: 16	(1994, 16)
MaxPooling 0	Pooling window: 2	(997, 16)
Conv 1	Kernel size: 5 Filters: 32	(993, 32)
MaxPooling 1	Pooling window: 2	(496, 32)
Conv 2	Kernel size: 3 Filters: 64	(494, 64)
Conv 3	Kernel size: 3 Filters: 128	(492, 128)
MaxPooling 2	Pooling window: 2	(246, 128)
Conv 4	Kernel size: 3 Filters: 256	(244, 256)
Conv 5	Kernel size: 3 Filters: 512	(242, 512)
Conv 6	Kernel size: 3 Filters: 1024	(240, 1024)
MaxPooling 3	Pooling window: 2	(120, 1024)
Fully connected		(256)
Sigmoid		(1)

### Effect of improved date augmentation on results

**Figure 4:**
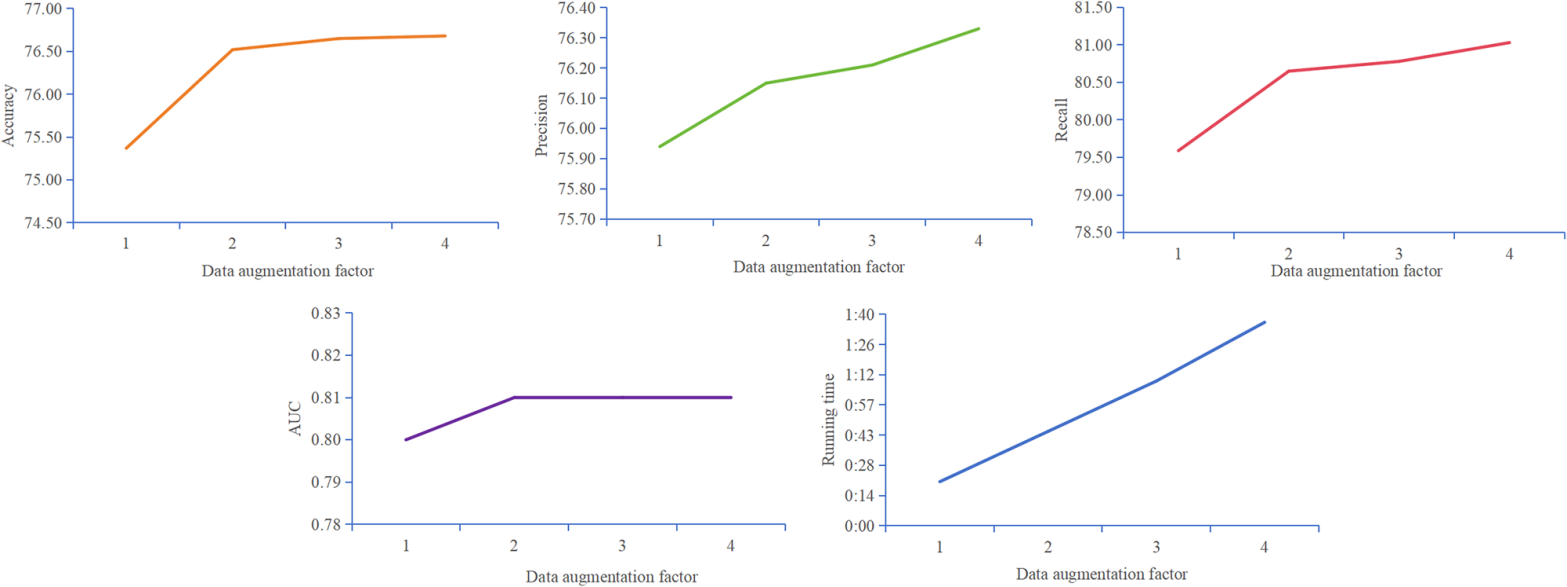
Effects of data augmentation on model performance.

To verify the impact of the data augmentation algorithm on model performance, we performed nested ten-fold crossover on ASD-SWNet under the conditions of data augmentation factor $\gamma = 1$ (no data augmentation), $\gamma = 2$, $\gamma = 3$, and $\gamma = 4$ respectively. verify. Figure 4 shows the change in model performance with increasing levels of data augmentation.

With a data augmentation factor of $\gamma = 1$, the model’s accuracy reached 75.37%, with an AUC of 0.80. Without the use of data augmentation, our model demonstrated high performance. At $\gamma = 2$, the model's accuracy reached 76.52%, with an AUC of 0.81, indicating a high overall performance. As we continued to enhance the dataset, at $\gamma = 3$ and $\gamma = 4$, the model’s accuracy, precision, and recall all improved. Compared to methods not utilizing data augmentation, at $\gamma = 4$, the model’s accuracy improved by 1.31%, precision by 0.39%, recall by 1.44%, and AUC by 1%. The significant enhancement in model performance adequately demonstrates the effectiveness of our proposed data augmentation algorithm.

From Fig. 4, it can be observed that the greatest improvement in model performance occurs at $\gamma = 2$. As the data augmentation factor increases, the extent of improvement in model performance becomes limited. At $\gamma = 3$, the model's accuracy improved by 0.13%. At $\gamma = 4$, the accuracy increase was merely 0.03%. Such marginal growth is difficult to classify as a breakthrough in model performance. This may be attributed to excessive enhancement and increased model complexity. Overutilization of data augmentation may lead to model overfitting, where excessive data variability causes the model to learn noise rather than meaningful patterns. Considering the running time, we noted that with the increase in the data augmentation factor, the running time showed a trend of nearly proportional growth. This is not conducive to the model’s training. Therefore, combining all performance metrics and running time, we believe that the model achieves optimal performance at $\gamma = 2$.

Reflecting on Fig. 4, the enhanced data augmentation’s impact on ASD-SWNet underscores our commitment to advancing ASD diagnostics through innovative machine learning techniques. By achieving a model accuracy of 76.52% and an AUC of 0.81 with an augmentation factor of 2, we not only address the challenge of data scarcity and imbalance but also enhance the model’s robustness and generalizability. This advancement directly aligns with our core motivation: to develop a reliable, efficient tool for the early and accurate diagnosis of autism spectrum disorder. Our contributions are twofold: introducing a novel data augmentation strategy that significantly improves diagnostic performance, and, more broadly, pushing the boundaries of how machine learning can be applied in medical image processing to benefit clinical practices and patient outcomes.

### Performance evaluation

Our proposed method, ASD-SWNet, was evaluated by comparing it against baseline and state-of-the-art (SOTA)^43^ models on the ABIDE-I dataset. Unless specified, our experiments were carried out under the condition $\gamma = 2$ data augmentation. SVM^44^, HOFC^45^, and GCN^46^ were used as baselines, while recent studies such as ASD-DiagNet^32^, ASD-SAENet^47^, and Hi-GCN^48^ were considered SOTA models. It is noteworthy that to ensure consistency in experimental conditions, we used nested ten-fold cross-validation instead of the traditional approach. This method of cross-validation involved re-partitioning the dataset during model replication, with the newly partitioned test sets separated from the model training and feature selection processes. Nested cross-validation provides a more reliable and unbiased performance estimate for the model.

ASD-DiagNet implements a joint learning process utilizing autoencoders and single-layer perceptrons to enhance the quality of feature extraction and optimize model parameters. ASD-SAENet calculates functional connectivity between brain regions using the Pearson correlation coefficient and performs classification using sparse autoencoders. Hi-GCN, on the other hand, concurrently considers the internal structure of individual brain functional networks and the structure of the entire population network, effectively learning high-level embedding representations of brain networks. The Hi-GCN framework comprises two separate graph convolutional networks (GCNs), each designed to model individual brain functional networks and the overall population graph network, respectively. The graph-level embedding learning of individual brain functional networks employs a GCN named f-GCN, while the graph-level embedding learning for the entire population network utilizes another GCN, termed p-GCN. During the training of p-GCN, graph kernels are used to measure the similarity between brain networks. The joint learning approach of Hi-GCN effectively facilitates the learning of high-level embedding representations of brain networks.

Table 4 displays the classification results on the ABIDE-I dataset. It can be observed that the ASD-SWNet method achieved the highest performance with an accuracy of 76.52%, precision of 76.15%, recall of 80.65%, and an AUC of 0.81. The network with shared weights effectively combines unsupervised and supervised learning methods, endowing the model with enhanced classification capabilities.

**Table 4:** Performance of our method and existing models.

Method	Accuracy	Precision	Recall	AUC	p-value
SVM	67.30	67.41	65.01	0.74	< 0.01
HOFC	71.13	68.97	61.03	0.77	
GCN	70.58	68.32	74.29	0.72	
ASD-DiagNet	69.07	65.25	69.58	0.71	
ASD-SAENet	71.05	72.67	64.76	0.73	
Hi-GCN	72.63	65.16	70.54	0.79	
ASD-SWNet	**76.52**	**76.15**	**80.65**	**0.81**	

Significant values are given in bold.

SVM is capable of extracting useful features from data for classification, yet it exhibits relatively poor performance. HOFC achieves a high performance with 71.13% accuracy and an AUC of 0.77. This indicates that, compared to FC, higher-order functional connectivity can more sensitively detect population differences and better capture individual variations. Our proposed method still manages to improve accuracy by 5.39% and AUC by 4%. The GCN approach uses non-imaging data of patients as a supplement, allowing the model to learn more meaningful features. However, due to the limitations of GCN, with shallow model layers unable to learn deeper features, it only achieves an accuracy of 70.58% and an AUC of 0.72.

Among all SOTA methods, Hi-GCN achieved the best performance with an accuracy of 72.63% and an AUC of 0.79, followed by ASD-SAENet with an accuracy of 71.05% and an AUC of 0.73. The lowest-performing SOTA model was ASD-DiagNet, with only 69.07% accuracy and 0.71 AUC. ASD-DiagNet uses an AE and an SLP for feature extraction and classification, sharing losses between AE and SLP, which leads to lower model complexity and effectively reduces overfitting while enhancing model generalizability. ASD-SAENet also shares losses but differs from ASD-DiagNet by using a DNN as the classifier. The multi-layer feature representation of ASD-SAENet results in superior performance, indicating the significant impact of appropriate model architecture even in tasks with smaller datasets. Hi-GCN’s two-level GCN effectively utilizes both imaging and non-imaging data, demonstrating the efficacy of joint learning in deriving structural brain region information and aggregating embeddings of adjacent topics to learn advanced brain network representations. Our proposed ASD-SWNet showed an improvement of 3.89% in accuracy and 2% in AUC. This highlights the effectiveness of combining unsupervised feature extraction in weight-shared networks with supervised classification methods, along with the crucial role of data augmentation algorithms in enhancing model performance. Additionally, we employed student’s *t*-tests for statistical comparison between our method and others, setting the significance level $\alpha = 0.01$ to ascertain statistical significance. As shown in Table 4, all p-values were less than 0.01. Rigorous statistical analysis confirms the significant advantages of our proposed method.

Table 4 illustrates that ASD-SWNet outperforms baseline and state-of-the-art models, achieving an accuracy of 76.52%, precision of 76.15%, recall of 80.65%, and an AUC of 0.81 on the ABIDE-I dataset. This marked improvement emphasizes the effectiveness of our shared-weight mechanism and the synergy between unsupervised feature extraction and supervised learning classification. Our method’s superiority not only reaffirms the motivations behind this work—to address the pressing need for accurate, reliable ASD diagnostics through innovative machine learning techniques—but also highlights its significant contributions to the field. By enhancing the precision and reliability of ASD diagnosis, ASD-SWNet stands as a testament to the potential of advanced computational models in overcoming the limitations of current diagnostic methodologies, offering new avenues for early intervention and treatment planning. This analysis underscores the model’s innovative approach to leveraging machine learning for significant clinical impact, marking a pivotal contribution to the ongoing efforts to improve ASD diagnostic practices.

To verify the robustness of ASD-SWNet, we employed LOOCV on the ABIDE-II dataset. We selected topical research from the last three years on ABIDE-II as comparative experiments. AL-NEGAT^49^ is an adversarial learning-based node-edge graph attention network. It utilizes a novel attention-based adjacency matrix to perform graph convolution operations on these features. The model also adopts adversarial training methods to enhance its robustness and generalizability. STCAL^50^ includes a sliding cluster attention (SCA) module and a guided co-attention (GCA) module. The SCA module is used to extract dynamic local feature representations, while the GCA module is employed for joint learning of spatio-temporal attention representations. Based on SCA and GCA, a co-attention learning network is further established to perform feature representation and fusion. Finally, classification tasks are realized by linking a simple attention-aware classification network.

**Figure 5:**
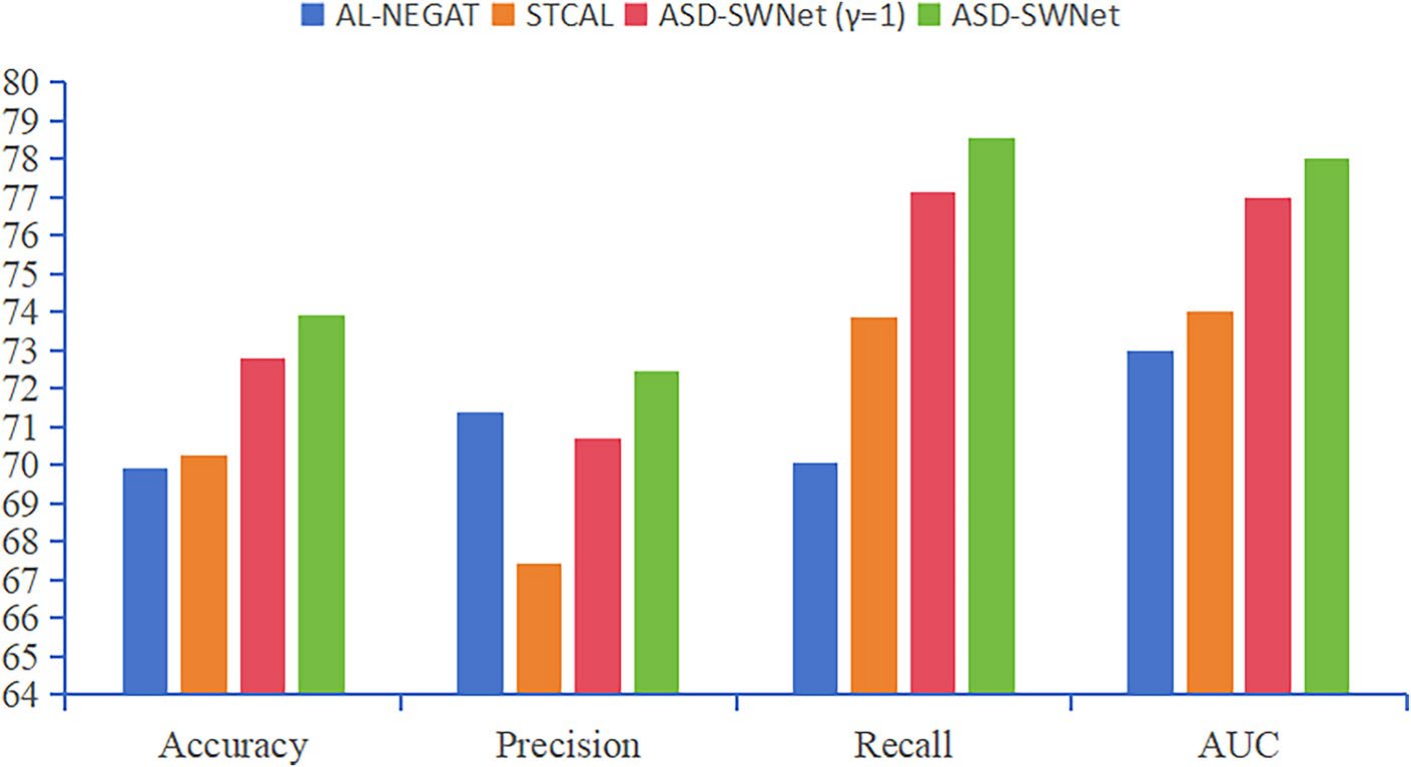
Results of different models on ABIDE-II.

Results from Fig. 5 demonstrate that ASD-SWNet outperforms all performance metrics on the ABIDE-II dataset. AL-NEGAT, which integrates node and edge features into graph classification tasks, showed improved classification performance with an accuracy of 69.91% and an AUC of 0.73. The adoption of adversarial training methods effectively enhanced the model’s robustness and generalizability. This result underscores the effectiveness of graph network-based approaches for fusing multimodal information to define graph features, leveraging both structural and functional information. STCAL achieved an accuracy of 70.25% and an AUC of 0.74, attributable to the self-attention mechanism providing new insights for dynamically detecting key time frames in time-series fMRI data. STCAL dynamically captures crucial time frames and spatio-temporal correlations in time-series fMRI data, thus improving diagnostic accuracy for psychiatric disorders. ASD-SWNet, even without data augmentation (${\upgamma = 1}$), already surpasses the other two SOTA methods. Compared to AL-NEGAT, ASD-SWNet (${\upgamma = 1}$) shows a 2.89% increase in accuracy, highlighting the superiority of our proposed method combining unsupervised feature extraction in a weight-shared network with supervised CNN classification. With the application of data augmentation algorithms, ASD-SWNet reached an accuracy of 73.92%, a precision of 72.45%, a recall of 78.53%, and an AUC of 0.78. The data augmentation algorithm increased the sample size, addressed imbalanced data, effectively reduced the risk of overfitting, and enhanced the model’s performance and robustness.

### Leave-one-site-out cross validation

To verify the model’s cross-site capability, we executed leave-one-site-out cross-validation^51^ under the condition of no data augmentation. First, we grouped the 871 samples from the ABIDE-I dataset by site, obtaining a total of 17 sites. Secondly, for each site, we used its data as the validation set, while the data from the other sites were combined to form the training set. Thereafter, we trained the model and validated it at each site. This process was repeated until each site had been validated once. Table 5 reports the accuracy in comparison with other methods.

**Table 5:** Performance of our method and existing models.

Site	Size	GCN	ASD-DiagNet	ASD-SAENet	ASD-SWNet
CALTECH	15	56.37	52.13	56.67	**70.91**
CMU	11	71.12	68.52	70.63	**78.76**
KKI	33	70.96	69.32	71.85	**75.12**
LEUVEN	56	63.72	61.54	64.36	**73.88**
MAX_MUN	46	49.31	47.76	48.02	**67.36**
NYU	172	71.04	67.94	71.96	**77.09**
OHSU	25	70.46	**81.76**	72.11	75.31
OLIN	28	65.06	64.81	65.89	**79.27**
PITT	50	70.51	66.98	**72.57**	71.46
SBL	26	57.01	51.63	57.54	**77.23**
SDSU	27	**65.96**	63.90	64.21	65.62
STANFORD	25	52.39	63.37	53.19	**67.45**
TRINITY	44	57.97	54.88	57.46	**63.57**
UCLA	85	67.07	**72.85**	68.39	70.02
UM	120	66.80	63.50	68.05	**79.41**
USM	67	70.05	69.01	**70.28**	65.76
YALE	41	65.19	62.86	66.74	**72.66**
Average	51.25	64.18	63.69	64.70	**72.40**

Significant values are given in bold.

We validated GCN^46^, ASD-DiagNet^32^, and ASD-SAENet^47^ on the CC200 atlas. The results show that our method performed better in terms of accuracy in 12 out of the 17 sites. ASD-SWNet achieved an average accuracy of 72.40%, surpassing the comparative methods. Consistent with the findings of Wang et al.^52^, the accuracy rates at sites such as MAX_MUN, SDSU, STANFORD, TRINITY, and USM were all below 70%. This indicates the presence of heterogeneity not found in data from other sites.

Our ASD-SWNet model showcases a superior cross-site capability, outperforming other models at the majority of sites. This achievement not only emphasizes the model’s adaptability and effectiveness across different datasets but also marks a significant contribution towards enhancing the precision and reliability of ASD diagnostics. In summary, ASD-SWNet outperforms other SOTA methods at more sites, demonstrating the superior cross-site capability and robustness of the model we proposed.

### Ablation study

Having verified the effectiveness of the data augmentation algorithm, we next designed ablation experiments^53^ to validate other modules proposed in the study. Ablation experiments systematically dismantle various components of the model, facilitating a better verification of the ASD-SWNet’s effects and allowing for an understanding of its internal mechanisms, constituent elements, and contributions. Table 6 presents the results of the ablation experiments.

**Table 6:** Results of ablation experiment.

Method	Accuracy	F1-score	AUC
CNN	73.29	75.43	0.78
CNN + DAE	74.86	76.35	0.79
CNN + DAE + shared weights	76.52	78.16	0.81

CNN, with its inherent strong feature extraction capability, achieved an accuracy of 73.29%, an F1-score of 75.43%, and an AUC of 0.78 when solely responsible for feature extraction and classification. This indicates that the CNN designed in this study demonstrates robust classification ability for ASD data, validating the effectiveness of CNN. Building on this, we first utilized DAE to extract low-dimensional features from the data, then input these preliminarily extracted features into our designed CNN for further feature extraction and completion of the classification task. After processing by DAE, the model’s accuracy improved by 1.57%, the F1-score by 0.92%, and the AUC by 1%, indicating the beneficial impact of DAE’s low-dimensional feature processing on the model’s performance. Furthermore, sharing weights between DAE and CNN during training, as seen in the results from Table 6, led to a 1.88% increase in accuracy, a 1.81% increase in F1-score, and an approximate 2% increase in AUC compared to methods without weight sharing. This suggests that the weight-sharing approach aids the model in effectively identifying more meaningful features, thereby enhancing performance. AE can learn effective feature representations of data, which CNN can leverage. Additionally, AE helps in learning redundant information in the data, enabling CNN to reduce redundant feature representations. Finally, AE, through nonlinear mapping, learns complex relationships in data, which CNN can utilize to better handle image data. In summary, in our designed weight-shared model, CNN benefits from the feature representations learned by AE, eliminating the need to learn these features from scratch, and thereby improving model performance. Ablation experiments indicate that each component of our proposed method enhances the model's performance and contributes to its robustness.

This systematic component evaluation aligns perfectly with our motivation to develop a robust, accurate diagnostic tool for ASD that leverages the full potential of machine learning. The ablation study not only demonstrates the individual contributions of CNN, DAE, and shared weights but also collectively emphasizes the innovative approach of integrating these components to improve diagnostic accuracy. This methodological advancement contributes significantly to the field of ASD diagnosis, showcasing a novel, effective way to address the challenges of ASD classification using machine learning techniques.

### Visualization

**Figure 6:**
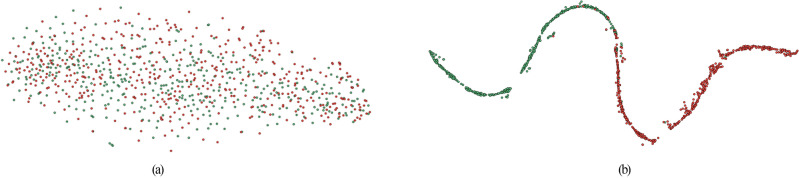
2D space visualization results. Green nodes denote autism spectrum disorder and red nodes denote healthy controls. (**a**) 2D Space visualization of original features; (**b**) 2D Space visualization following ASD-SWNet features.

We employed the t-SNE technique to visualize both the original data and the results post-classification by ASD-SWNet in a 2D space. This was done to observe the performance of our proposed method in terms of feature fusion and classification. The visualization results are illustrated in Fig. 6.

As shown in Fig. 6a, the original data distribution of ASD and HC is random and disordered, with no clear boundary between the two categories, indicating that classification is highly challenging. After classification by ASD-SWNet, the two-dimensional feature visualization of the classification results is presented in Fig. 6b. Here, the blue nodes are clustered together, showing a very tight distribution. The orange nodes display a similar distribution pattern. This suggests that the features post-ASD-SWNet classification exhibit good intra-class cohesion. Furthermore, the subgraphs corresponding to the blue and orange nodes are overall similar, indicating that the method distinctly separates ASD patients from the healthy control group, which means the classified features have notable inter-class discriminability. The data visualization results thoroughly demonstrate the exceptional classification capability of our proposed ASD-SWNet model.

## Conclusion

This study proposes an autism spectrum disorder diagnostic method based on a shared weights network. It incorporates a data augmentation algorithm, an unsupervised learning feature extraction method, and a supervised learning convolutional neural network classification approach. The data augmentation algorithm addresses the issues of insufficient data leading to inadequate model training and overfitting. Utilizing an AE to extract low-dimensional features from the original data and performing preprocessing, these features are then input into the CNN to further extract high-dimensional features for classification tasks. Notably, this study achieves weight sharing between AE and CNN, linking the entire model together, enabling the CNN to extract more meaningful features, enhancing the performance of the classification model, and effectively combining unsupervised and supervised learning. Compared to existing methods, our proposed approach achieves the highest performance in identifying ASD and offers a broad prospect for clinical application. Lastly, this research also has certain limitations; we did not consider the supplementary role of non-imaging data, such as patient age, gender, and family history, as would be queried by a clinician. In the future, we plan to use graph neural networks to combine non-imaging data to explore its impact on model performance. Graph edges can effectively encode associations between nodes, offering more possibilities for multimodal ASD diagnosis.

## Data Availability

The data used in this article can be found at the following link: https://fcon_1000.projects.nitrc.org/indi/abide/.
